# Current and novel biomarkers in cardiogenic shock

**DOI:** 10.1002/ejhf.3531

**Published:** 2025-01-17

**Authors:** Victor Galusko, Florian A. Wenzl, Christophe Vandenbriele, Vasileios Panoulas, Thomas F. Lüscher, Diana A. Gorog

**Affiliations:** ^1^ Royal Brompton and Harefield Hospitals, Guy's and St. Thomas' NHS Foundation Trust London UK; ^2^ Centre for Molecular Cardiology University of Zurich Schlieren Switzerland; ^3^ National Disease Registration and Analysis Service NHS London UK; ^4^ Department of Cardiovascular Sciences University of Leicester Leicester UK; ^5^ Department of Clinical Sciences Karolinska Institutet Stockholm Sweden; ^6^ Heart Center OLV Hospital Aalst Belgium; ^7^ Faculty of Medicine, National Heart and Lung Institute Imperial College London UK; ^8^ School of Cardiovascular Medicine and Sciences Kings College London London UK; ^9^ School of Life and Medical Sciences, Postgraduate Medical School University of Hertfordshire Hertfordshire UK

**Keywords:** Acute myocardial infarction, Acute heart failure, Cardiogenic shock, Biomarkers, Mechanical circulatory support

## Abstract

Cardiogenic shock (CS) carries a 30–50% in‐hospital mortality rate, with little improvement in outcomes in the last decade. Challenges in improving outcomes are closely linked to the frequent late presentation or diagnosis of CS where the ‘point of no return’ has often passed, leading to haemodynamic dysregulation, progressive myocardial depression, hypotension, and a downward spiral of hypoperfusion, organ dysfunction and decreasing myocardial function, driven by inflammation and metabolic derangements. Novel therapeutic interventions may have varying efficacy depending on the type and stage of shock in which they are applied. Biomarkers that aid prediction and early detection of CS, provide early signs of organ dysfunction and define prognosis could help optimize management. Temporal change in such biomarkers, particularly in response to pharmacological interventions and/or mechanical circulatory support, can guide management and predict outcome. Several novel biomarkers enhance the prediction of mortality in CS, compared to conventional parameters such as lactate, with some, such as adrenomedullin and circulating dipeptidyl peptidase 3, also able to predict the development of CS. Some biomarkers reflect systemic inflammation (e.g. interleukin‐6, angiopoietin 2, fibroblast growth factor 23 and suppressor of tumorigenicity 2) and are not specific to CS, yet inform on the activation of important pathways involved in the downward shock spiral. Other biomarkers signal end‐organ hypoperfusion and could guide targeted interventions, while some may serve as novel therapeutic targets. We critically review current and novel biomarkers that guide prediction, detection, and prognostication in CS. Future use of biomarkers may help improve management in these high‐risk patients.

## Introduction

Cardiogenic shock (CS) remains a major cause of morbidity and mortality in patients with acute coronary syndrome (ACS) and acute heart failure (HF).^1^ Traditionally defined as ineffective cardiac output due to primary cardiac dysfunction, resulting in inadequate end‐organ perfusion, the Society for Cardiovascular Angiography and Interventions (SCAI) has more recently delineated different gradations of CS.^2^, ^3^

The high mortality rate associated with CS has not improved significantly over the last few decades.^4^, ^5^ In‐hospital mortality remains at 30–50%, and of those patients surviving until discharge, about 48% are readmitted and 15% die within a year.^4^, ^6^ Treatment options are limited, and plagued by a failure of treatments to disrupt the complex downward spiral, once CS sets in.^3^, ^7^

The pathophysiological mechanisms underlying the shock spiral are incompletely understood, although dysfunctional regulatory pathways are likely involved. Endothelial responses that are initially protective become harmful. Vasoconstriction, initiated by activation of the sympathetic nervous system, is eventually reversed by β‐adrenergic receptor downregulation and inflammation, due to activation of nucleotide‐binding oligomerization domain‐like receptor protein 3 inflammasome and interleukins (IL), leading to inappropriate vasodilatation. Expression of inducible nitric oxide synthase results in an increase in levels of nitric oxide and reactive oxygen species, leading to the production of cytotoxic products.^8^ This results in negative ionotropic effects, suppression of mitochondrial function, inflammation and reduced catecholamine responses, ultimately leading to vasodilatation.^9^

Biomarkers have emerged as indicators of poor outcome and potential novel therapeutic targets. In order to incorporate novel biomarkers into early identification, monitoring, prognostication and treatment of patients with CS, it is necessary to understand their origins, the biological pathways and interactions, and their time course in CS. Previous efforts to summarize biomarkers in CS have been brief, often lacking detailed exploration of all biomarkers, their pathophysiological mechanisms, and comparative diagnostic usefulness.^10^, ^11^, ^12^ Furthermore, an overview of the usefulness of these biomarkers in the context of mechanical circulatory support (MCS) is not described. Here, we aim to bridge this gap by summarizing current and novel biomarkers, discussing their merits and limitations, and identifying potential future avenues for research.

## Currently available biomarkers

Biomarkers are frequently incorporated into clinical scores for the risk stratification of patients with CS. Over 30 risk scores are currently available and incorporate a number of plasma biomarkers, including pH, lactate, creatinine, liver function tests, brain natriuretic peptide (BNP) and N‐terminal pro‐B‐type natriuretic peptide (NT‐proBNP).^13^, ^14^ All these markers are associated with increased mortality in Yes CS (*Table* *1*). Many also feature in the SCAI risk stratification system, designed to predict CS outcomes and aid inter‐hospital communication.^2^

**Table 1:** Current biomarkers used in the risk stratification of cardiogenic shock

Current biomarkers	Studies + (patient *n*=)	Aetiology of CS	Highest AUC for mortality (95% CI)	Investigated timing of biomarker	Cardiac specific?	Goes up in sepsis?	Goes up in malignancy?	Predicts onset of CS	Predicts mortality
pH [H+]^15^	Studies = 1 CS Patients = 1782	ACS, HF	‐	On admission	No	Yes	No	N/A (potentially^16^)	Yes
Lactate^15^, ^17^, ^18^, ^19^, ^20^, ^21^, ^22^, ^23^, ^24^, ^25^, ^26^	Studies = 12 CS Patients = 3912	ACS, HF, valvular, post‐cardiotomy	30‐day mortality = 0.69	On admission	No	Yes	Yes^27^	Yes	Yes
			30‐day mortality = 0.76	At 8‐h					
			30‐day mortality = 0.81	At 24‐h					
			In hospital mortality = 0.79	Lactate clearance at 24 h					
Glucose^28^, ^29^, ^30^, ^31^, ^32^	Studies = 5 CS Patients = 2555	ACS, HF	30‐day mortality = 0.65	On admission	No	Yes^1^	Yes^2^	N/A	Yes
Creatinine^33^ ^,^ ^a^	Studies = 1 CS Patients = 190	ACS	30‐day mortality and/or RRT = 0.79	Day 3	No	Yes	Yes	N/A	AKI is a strong risk factor for mortality
			1‐year mortality = 0.80	Day 3					
Troponin^34^, ^35^	Studies = 2 CS Patients = 258	ACS, HF, valvular, post‐cardiotomy	30‐day mortality = 0.67 (0.60–0.74)	On admission	Yes	Yes^36^	Yes^37^	Yes	Yes
Procalcitonin^38^, ^39^, ^40^, ^41^, ^42^	Studies = 5 CS Patients = 753	ACS, myocarditis	30‐day mortality = 0.66	On admission	No	Yes^43^	Yes^44^	N/A	Yes
			30‐day mortality = 0.85	At 24‐h					
proBNP^34^, ^38^, ^45^, ^46^, ^47^	Studies = 4 + 1 CS Patients = 410 + 7290	ACS (*n* = 306) HF (*n* = 7290)	30‐day mortality = 0.561	On admission	Yes	Yes^48^	Yes^49^	N/A	Yes
			30‐day mortality = 0.71	At 24‐h					

Current biomarkers	Correlates with end‐organ dysfunction in CS							MCS		
	Brain	Heart	Lung	Renal	Liver	Intestine	Haematologic/ bleeding	Predicts mortality on ECMO/MCS	Predicts weaning off ECMO	Predicts weaning off MCS
pH [H+]^15^	N/A (potentially^50^)	Yes	N/A	Yes	Yes	Yes	N/A (potentially^51^)	Yes^52^	N/A (potentially^53^	N/A
Lactate^17^	Yes	Yes	N/A (potentially^54^)	N/A (potentially^55^)	N/A (potentially^56^)	N/A (potentially^57^)	N/A (potentially^58^)	Yes^52^	N/A (potentially^53^)	Yes
Glucose^28^	N/A	Yes	N/A	N/A (potentially^3^)	N/A (potentially^4^)	N/A	N/A (potentially^5^)	Yes	N/A	N/A
Creatinine^33^ ^,^ ^a^	N/A	N/A	N/A	Yes	N/A	N/A	N/A	N/A	N/A	N/A
Troponin^34^	N/A	Yes	N/A	N/A (likely^59^)	N/A (potentially z^60^)	N/A	N/A	Yes	Yes^61^	N/A
Procalcitonin^40^	N/A	Yes	N/A	N/A (potentially^62^)	N/A (potentially^63^)	N/A (potentially^64^)	Yes	Yes	N/A	N/A
proBNP^38^	N/A	Yes	N/A (potentially^65^)	N/A (potentially^66^)	N/A	N/A	N/A (potentially^67^)	No	Unlikely^35^	N/A

The table summarizes their predictive abilities in CS, their correlation with end‐organ dysfunction and whether their use has been studied in the context of MCS. As concurrent diseases (such as sepsis and malignancy) may confound CS state, some of the biomarkers also increase in these conditions. The table is colour coded according to whether this information is available –. 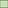, Yes; 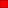, No; 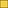, No evidence available.

ACS, acute coronary syndrome; AKI, acute kidney injury; AUC, area under the curve; CI, confidence interval; CS, cardiogenic shock; ECMO, extracorporeal membrane oxygenation; HF, heart failure; LC, lactate clearance; MCS, mechanical circulatory support; N/A, not available; NRI, net reclassification improvement; proBNP, pro‐B‐type natriuretic peptide.

^a^ Although creatinine is frequently used in the definition of AKI, which is a risk factor for mortality in CS, few studies examine the direct effect of creatinine on mortality in CS. A comprehensive review of AKI in CS can be found here.^68^

Lactate and pH are markers of tissue hypoperfusion resulting from anaerobic respiration and eventually tissue acidosis. Although lactate is highly predictive of in‐hospital mortality and readily available as a point‐of‐care test, it is not specific for CS, being also elevated as a consequence of impaired clearance, as seen in liver dysfunction and chronic kidney disease (CKD), drug interactions (e.g. metformin), or as a consequence of administration of high‐dose catecholamines.^69^ Lactate clearance may be a better predictor of mortality than circulating lactate level, especially in patients on MCS.^17^, ^69^, ^70^ Other studies indicated that an 8‐h lactate is superior to lactate clearance for the prediction of 30‐day mortality in CS.^18^, ^19^ The optimal time to measure lactate to predict prognosis remains unclear, especially as a single 8‐h value likely reflects lactate clearance. The prognostic value of lactate may decline over time, and is potentially affected by ionotropic and renal replacement therapies.^19^, ^71^ Despite these limitations, lactate is used routinely in the assessment of CS.

Both BNP and NT‐proBNP are elevated in CS, due to increased left ventricular (LV) filling pressures and are highly predictive of hospitalization and mortality.^72^, ^73^ Despite being included in several risk scores, the evidence supporting the usefulness of BNP in CS is mixed. Indeed, in the Interagency Registry for Mechanically Assisted Circulatory Support (INTERMACS), a large registry of patients with end‐stage and acute HF treated with LV assist device (LVAD) support, BNP was elevated, but did not correlate with mortality or major adverse cardiovascular events (MACE).^45^ Both BNP and NT‐proBNP levels are affected by renal clearance, accounting for some of the observed variability.^66^ Importantly, in a head‐to‐head comparison, adrenomedullin (ADM), IL‐6 and procalcitonin (PCT) all outperformed BNP in predicting CS mortality.^38^, ^46^, ^74^

In a prospective study of 273 patients with CS, half of whom had ACS, elevated troponin levels, reflecting greater myocyte loss, were a better predictor of 30‐day mortality than NT‐proBNP,^34^ and are associated with unsuccessful weaning from veno‐arterial extracorporeal membrane oxygenation (V‐A ECMO).^34^, ^61^, ^75^

The systemic inflammation that ensues in CS leads to an increase in white cell count and cytokine release.^76^ In patients with coronary artery disease, raised PCT levels are associated with MACE, and are a better predictor of all‐cause mortality in CS than C‐reactive protein (CRP).^38^, ^76^, ^77^ Inflammation and stress can result in hyperglycaemia.^78^ While diabetes in itself is a risk factor for CS, impaired fasting glucose and raised random glucose levels are adverse markers only in patients without a prior history of diabetes.^28^, ^29^

Current biomarkers are widely available and in use, but are non‐specific and have limitations, namely low sensitivity and specificity (*Table* *1*).

## Novel biomarkers

**Figure 1:**
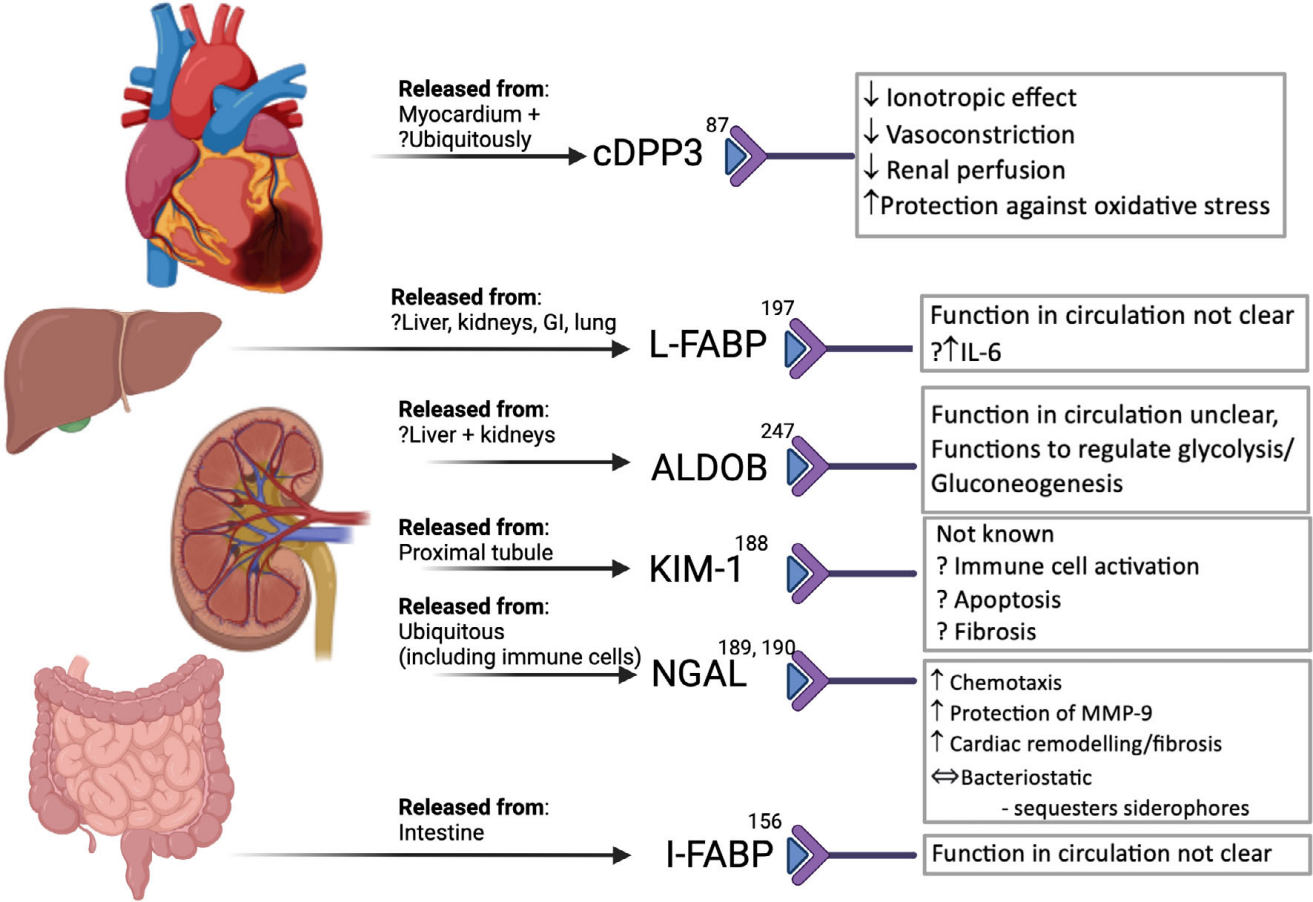
Summary of molecules released as a consequence of cellular damage in cardiogenic shock and their potential downstream effects in the circulation. Tissue injury occurs as a consequence of ischaemia and results in the release of molecules that have been associated with poor prognosis in cardiogenic shock. They have differing roles in the circulation, however we only have a limited understanding of their effects, especially in cardiogenic shock. cDPP3 has emerged as an important prognostic molecule in cardiogenic shock recently, and in a mouse model, it has a direct negative effect on the myocardium and kidneys. ALDOB, aldolase B; cDPP3, circulating dipeptidyl peptidase 3; GI, gastrointestinal; I‐FABP, intestinal fatty acid binding protein; IL‐6, interleukin‐6; KIM‐1, kidney injury molecule‐1; L‐FABP, liver fatty acid binding protein; MMP‐9, matrix metalloproteinase‐9; NGAL, plasma neutrophil gelatinase‐associated lipocalin.

**Figure 2:**
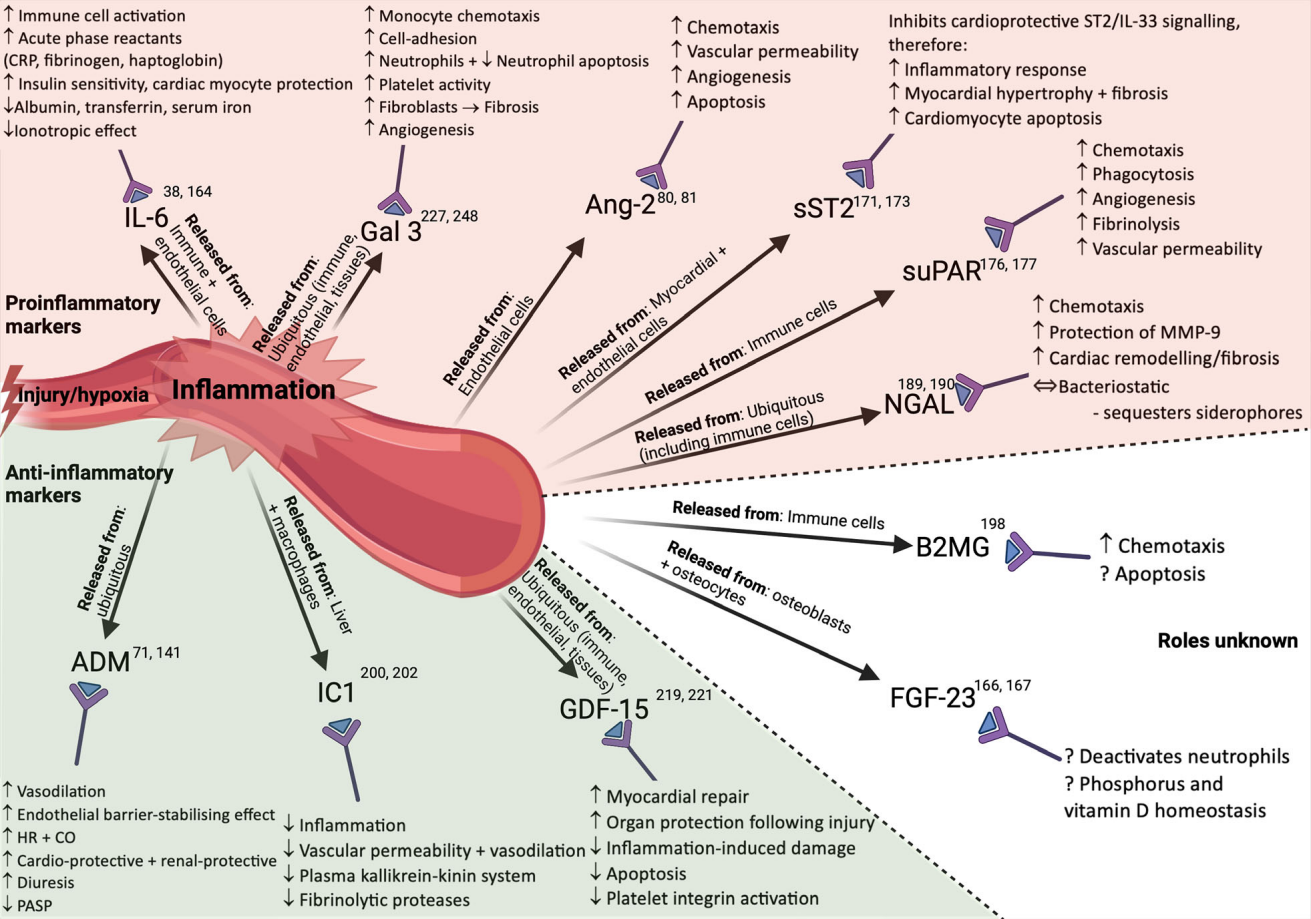
Summary of molecules released in response to inflammation in cardiogenic shock which act potentially in a proinflammatory and anti‐inflammatory fashion. Injury and hypoxia lead to inflammation that results in the release of a variety of molecules. Traditionally these have been associated with worsening hypoperfusion, ischaemia and end‐organ dysfunction. However, many molecules are likely to have differing effects depending on their target tissue and concentration, and while some are part of the immune system activation and lead to chemotaxis, fibrosis, and apoptosis, others act in a cardioprotective manner trying to promote healing, stabilization and limit inflammation. ADM, adrenomedullin; Ang‐2, angiopoietin‐2; B2MG, beta‐2 microglobulin; CO, cardiac output; CRP, C‐reactive protein; FGF‐23, fibroblast growth factor 23; Gal‐3, galectin 3; GDF‐15, growth differentiation factor 15; HR, heart rate; IC1, C1 inhibitor protein; IL‐6, interleukin‐6; IL‐33, interleukin‐33; MMP‐9, matrix metalloproteinase‐9; NGAL, plasma neutrophil gelatinase‐associated lipocalin; NRI, net reclassification improvement; PASP, pulmonary artery systolic pressure; sST2, soluble suppressor of tumorigenicity 2; suPAR, soluble urokinase‐type plasminogen activator receptor.

**Figure 3:**
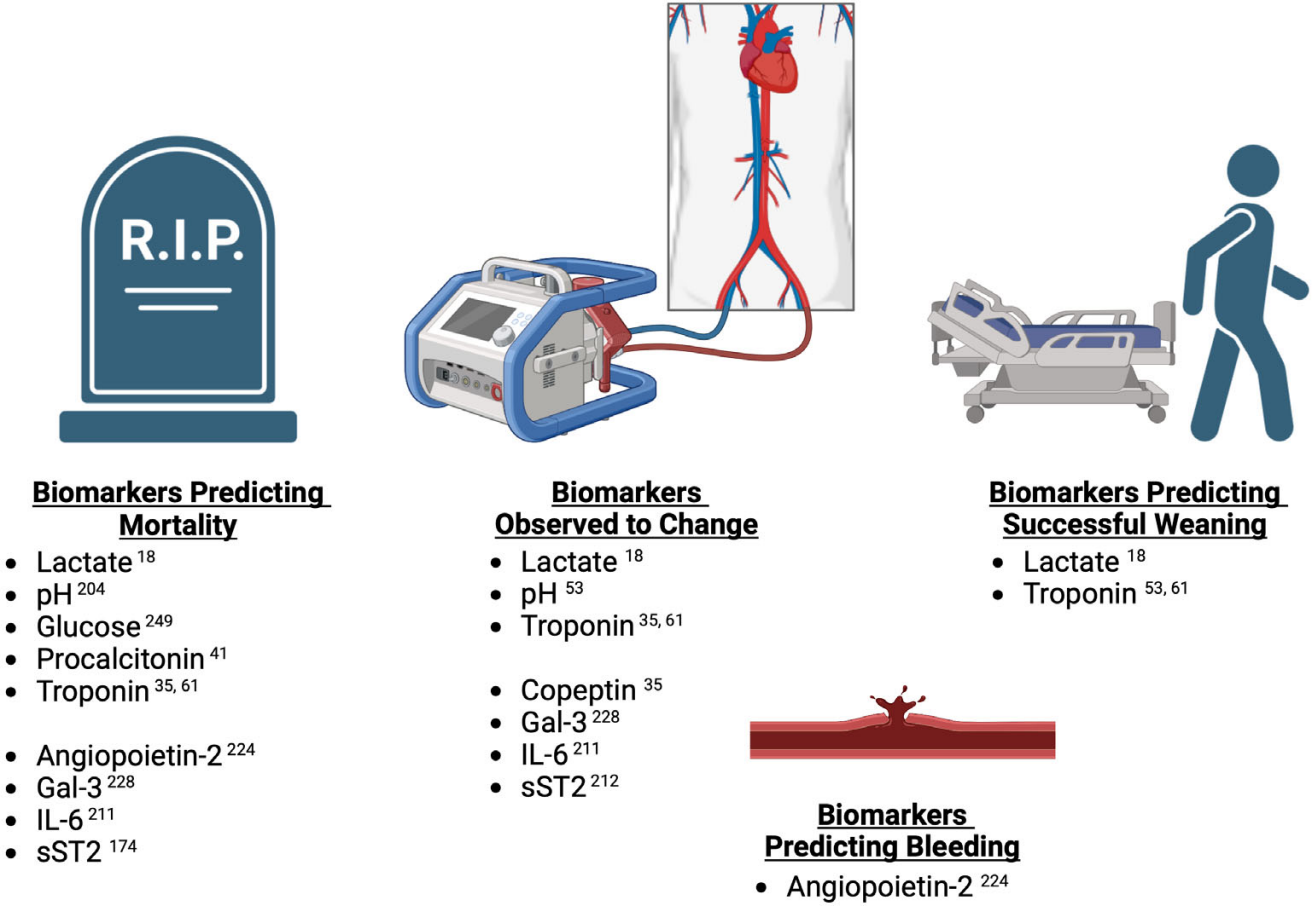
Summary of the biomarkers in patients on mechanical circulatory support (MCS) predicting mortality, seen to change on MCS, and biomarkers which predict successful weaning from MCS. A selection of biomarkers has been investigated in the context of MCS and while some predict mortality, others are seen to change in response to therapy and some may be useful in predicting the ability to wean MCS. Gal‐3, galectin 3; IL‐6, interleukin‐6; sST2, soluble suppressor of tumorigenicity 2.

A number of novel biomarkers, reflecting the dysfunction of different organ systems, are released into the circulation during the various phases of CS (*Graphical Abstract*). The physiological or pathological mechanisms through which novel biomarkers reflect the development of or influence the outcome of CS are not fully understood, although some have tried to shed light on this.^79^ Some biomarkers are released as a result of cellular injury (*Figure* *1*), while others are released as part of the inflammatory response that ensues (*Figure* *2*). Most of the biomarkers discussed below are measurable in blood plasma, unless stated otherwise, and are summarized in *Table* *2*. All these novel biomarkers can guide prognosis, but some also predict the onset of CS and mortality, while others forecast end‐organ dysfunction. As CS progresses and necessitates MCS, certain biomarkers have been shown to predict recovery, complications of, and ease of weaning from MCS (*Figure* *3*).

**Table 2:** Novel biomarkers studied in risk stratification of cardiogenic shock

Potential future biomarkers	Studies + (patient *n*=)	Aetiology of CS	Highest AUC for mortality (95% CI)	Investigated timing of biomarker	NRI available	External validation	Cardiac specific?	Goes up in sepsis?	Goes up in malignancy?	Predicts onset of CS	Predicts mortality
Individual markers											
Ang‐2^80^	Studies = 3 CS Patients = 323	ACS, HF	28‐day mortality = 0.71^81^	Day of admission to ICU	No	Yes	No	Yes^82^	Yes^83^	N/A	Yes
			1‐year mortality = 0.71^80^	Day of admission (within 12 h of CS onset)							
Co‐peptin^84^	Studies = 1 CS Patients = 225	ACS	N/A	On admission to cath lab	No	No	No	Yes^85^	Unlikely^205^	Yes^84^	N/A
cDPP3^87^	Studies = 3 CS Patients = 166	ACS, HF	90‐day mortality = 0.60	At ‘onset’ of CS	No	Yes	No	Yes^88^	Yes^89^	Yes	Yes
Gal‐3^90^	Studies = 1 CS Patients = 40	ACS	30‐day mortality = 0.70 (0.51–0.88)	At admission to hospital	No	No	No	Yes^91^	Yes^92^	N/A	Yes
GDF‐15^93^	Studies = 2 CS Patients = 367	ACS	90‐day mortality = 0.70 (0.62–0.77)	At ‘onset’ of CS	Yes	Yes	No	Yes^94^	Yes^95^	N/A	Yes
			90‐day mortality = 0.81 (0.74–0.88)	At 12 h following CS							
ADM^71^	Studies = 3 CS Patients = 277	ACS, HF	30‐day mortality = 0.71 (0.55–0.86)	At ‘inclusion’ defined as CS	No	Yes	No	Yes^96^	Yes^97^	Yes^84^	Yes
			90‐day mortality = 0.80 (0.78–0.91)	At 5–10 days							
proANP^84^	Studies = 1 CS Patients = 225	ACS	N/A	On admission to cath lab	No	No	Yes	Yes^98^	N/A	Yes	N/A
sST2^84^	Studies = 5 CS Patients = 402	Mainly ACS; HF	30‐day mortality = 0.55	At ‘inclusion’ defined as CS	No	No	No	Yes^99^	Yes^100^	Yes^84^	Yes
			30‐day mortality = 0.93	At 10 days							
I‐FABP^101^	Studies = 1 CS Patients = 90	ACS/HF	30‐day mortality = 0.72 (0.62–0.82)	At admission to ICU	No	No	No	Yes^102^	No	N/A	Yes
suPAR^103^	Studies = 1 CS Patients = 161	ACS	90‐day mortality = 0.72	At ‘inclusion’ with CS (within 6 h of CS onset)	No	No	No	Yes^104^	Yes^105^	N/A	Yes
Cystatin C^33^	Studies = 1 CS Patients = 190	ACS	1‐year mortality = 0.57	At admission to hospital	No	No	No	No^106^	Yes^107^	N/A	Yes
KIM‐1^33^	Studies = 1 CS Patients = 190	ACS	1‐year mortality = 0.60	At admission to hospital	No	No	No	Yes^108^	Yes^109^	N/A	Yes
P‐NGAL^33^	Studies = 2 CS Patients = 344	Mainly ACS	90‐day mortality = 0.78	At 24 h	No	Yes	No	Yes^110^	Yes^111^	N/A	Yes
			1‐year mortality = 0.62	At admission to hospital							
IL‐6^38^	Studies = 2 CS Patients = 270	ACS	30‐day mortality = 0.825	At admission to hospital	No	Yes	No	Yes^112^	Yes^113^	N/A	Yes
			90‐day mortality = 0.74 (0.66–0.82)	At 24 h							
FGF‐23^114^	Studies = 1 CS Patients = 51	ACS	28‐day mortality = 0.69	At admission to ICU	No	No	No	No^115^	Yes^116^	N/A	Yes
P‐PENK^117^	Studies = 1 CS patients = 154	ACS	90‐day mortality = 0.68	At ‘inclusion’ with CS (within 6 h of CS onset)	No	No	No	Yes^118^	N/A	N/A	Yes
			90‐day mortality = 0.76	At 24 h							
Combination scores											
CLIP (cystatin C, lactate, IL‐6, and NT‐proBNP)^119^	Studies = 1 CS Patients = 458	ACS	30‐day mortality = 0.83 (0.77–0.90)	On admission to cath lab	No	Yes	No	Yes	N/A	N/A	Yes
CS4P (L‐FABP, B2MG, ALDOB, and IC1)^120^	Studies = 1 CS Patients = 48	ACS	90‐day mortality = 0.83 (0.74–0.89)	Derivation: On admission – 24 h Validation: within 6 h of CS	Yes	Yes	No	N/A	N/A	N/A	Yes

Potential future biomarkers	Correlates with end‐organ dysfunction in CS							MCS		
	Brain	Heart	Lung	Renal	Liver	Intestine	Haematologic/ Bleeding	Predicts mortality on ECMO/MCS	Predicts weaning off ECMO	Predicts weaning off MCS
Individual markers										
Ang‐2^80^	N/A	Yes	N/A	Yes	N/A (potentially^121^)	N/A	Yes	Yes	N/A	N/A
Co‐peptin^84^	N/A	Yes	N/A	N/A (potentially^122^)	N/A	N/A	N/A	N/A	Unlikely^35^	N/A
cDPP3^87^	N/A	Yes	N/A	N/A (potentially^123^)	N/A	N/A	N/A	N/A	N/A	N/A
Gal‐3^90^	N/A	Yes	N/A (potentially^124^)	N/A (potentially^125^)	N/A (potentially^126^)	N/A	N/A (potentially^127^)	Yes	N/A (unlikely^128^)	N/A
GDF‐15^93^	N/A (potentially^129^)	Yes	N/A	N/A (potentially^130^)	N/A	N/A	N/A (potentially^131^)	N/A	N/A	N/A
ADM^71^	N/A	Yes	N/A	N/A (potentially^132^)	N/A	N/A	N/A	N/A	Unlikely^35^	N/A
proANP^84^	N/A	Yes	N/A	N/A	N/A	N/A	N/A	N/A	Unlikely^35^	N/A
sST2^84^	N/A	Yes	N/A	Yes	N/A (potentially^133^)	N/A	N/A	Yes	N/A (potentially^128^)	N/A
I‐FABP^101^	N/A	Yes	N/A	Yes	N/A	No (potentially^102^)	N/A	N/A	N/A	N/A
suPAR^103^	N/A (potentially^134^)	Yes	N/A	N/A (potentially^135^)	N/A (potentially^136^)	N/A	N/A	N/A	N/A	N/A
Cystatin C^33^	N/A	N/A	N/A	Yes	N/A	N/A	No^137^	N/A	N/A	N/A
KIM‐1^33^	N/A	Yes	N/A	Yes	N/A	N/A	N/A	N/A	N/A	N/A
P‐NGAL^33^	N/A	Yes	N/A	Yes	N/A	N/A	N/A	N/A	N/A	N/A
IL‐6^38^	N/A	Yes	N/A	Yes	N/A	N/A (potentially^138^)	N/A	Yes	N/A	N/A
FGF‐23^114^	N/A	Yes	N/A	N/A (potentially^139^)	N/A	N/A	N/A	N/A	N/A	N/A
P‐PENK^117^	N/A	Yes	N/A	Yes	N/A	N/A	N/A	N/A	N/A	N/A
Combination scores										
CLIP (cystatin C, lactate, IL‐6, and NT‐proBNP)^119^	N/A	Yes	N/A	N/A	N/A	N/A	N/A	N/A	N/A	N/A
CS4P (L‐FABP, B2MG, ALDOB, and IC1)^120^	N/A	Yes	N/A	N/A	N/A	N/A	N/A	N/A	N/A	N/A

The table summarizes their predictive ability in CS, their correlation with end‐organ dysfunction and whether their use has been studied in the context of MCS. As concurrent diseases, such as sepsis and malignancy, may confound the state of CS, it is important to consider whether the biomarkers change in response to these conditions and is included the in table. The table is colour‐coded according to whether this information is available ‐. 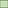, Yes; 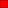, No; 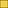, No evidence available.

ACS, acute coronary syndrome; ADM, adrenomedullin; ALDOB, aldolase B; Ang‐2, angiopoietin‐2; AUC, area under the curve; B2MG, beta‐2‐microglobulin; cDPP3, circulating dipeptidyl peptidase 3; CI, confidence interval; CS, cardiogenic shock; CS4P, cardiogenic shock 4 proteins; ECMO, extracorporeal membrane oxygenation; FGF‐23, fibroblast growth factor 23; Gal‐3, galectin 3; GDF‐15, growth differentiation factor 15; HF, heart failure; I‐FABP, intestinal fatty acid binding protein; IC1, C1 inhibitor protein; IL‐6, interleukin‐6; KIM‐1, kidney injury molecule‐1; L‐FABP, liver fatty acid binding protein; MCS, mechanical circulatory support; N/A, not available; NRI, net reclassification improvement; NT‐proBNP, N‐terminal pro‐B‐type natriuretic peptide; P‐NGAL, plasma neutrophil gelatinase‐associated lipocalin; P‐PENK, plasma proenkephalin; proANP, pro‐atrial natriuretic peptide; sST2, soluble suppressor of tumorigenicity 2; suPAR, soluble urokinase‐type plasminogen activator receptor.

## Novel biomarkers predicting onset of cardiogenic shock

### Adrenomedullin

Adrenomedullin (ADM), originally isolated from the adrenal medulla, is expressed by all tissues, including endothelial and smooth muscle cells and fibroblasts.^140^ ADM is a vasoactive peptide produced from a multistep proteolytic cleavage of prepro‐ADM. This process also generates adrenotensin and a pro‐ADM N‐terminal 20 peptide (PAMP), which themselves are vasoactive (PAMP exerts vasodilatory effects, whereas adrenotensin is vasoconstrictive).^141^ Myocardial stunning and ischaemia lead to increased LV filling pressures, triggering a neurohumoral response involving catecholamines, aldosterone and angiotensin II, that all further stimulate ADM production.^71^, ^142^ The inflammatory response that ensues (involving IL‐1 and tumour necrosis factor‐α) further stimulates ADM production.^143^

Besides being a powerful predictor of adverse outcome in patients with ACS and acute HF, ADM outperforms troponin, BNP and CRP in predicting 30‐day mortality.^142^, ^144^

However, in a recent substudy of the STRONG‐HF trial enrolling 1005 patients, both ADM and NT‐proBNP showed similar area under the curve (AUC) for predicting 180‐day all‐cause mortality or HF rehospitalization.^145^ In patients hospitalized with suspected ST‐elevation myocardial infarction (STEMI), the non‐biologically active precursor, mid‐regional pro‐ADM (MR‐proADM), was independently predictive of CS, with an AUC of 0.82.^84^ Biologically active ADM showed increasing prognostic value from 48 h post‐diagnosis and beyond, with AUC 0.71 at 48 h and 0.80 at 5–10 days.^71^ It has been used to predict 30‐day, 90‐day and 1‐year mortality in ACS patients with CS, and improves the prediction of CS when added to risk scores such as the Observatoire Régional Breton sur l'Infarctus (ORBI) or CardShock.^46^, ^71^, ^146^ ADM correlates with haemodynamic indices including reduced cardiac index, mean arterial pressure, central venous pressure, and systolic pulmonary artery pressure, alongside markers of end‐organ dysfunction.^71^ Furthermore, high ADM levels at or beyond 48 h correlate with persistently impaired LV function and decreased vasopressor responsiveness in sepsis.^71^, ^147^, ^148^

### Circulating dipeptidyl peptidase 3

Dipeptidyl peptidase 3 (DPP3) is a ubiquitously expressed enzyme, digesting several oligopeptides including angiotensins, enkephalins and endorphins.^149^ Upon cell death, this zinc‐dependent metallopeptidase is released into the circulation, where elevated levels have been measured in cardiogenic, septic and haemorrhagic shock.^150^

Elevated circulating DPP3 (cDPP3) levels are associated with increased mortality in CS and are predictive of the development of in‐hospital CS.^87^, ^151^ Addition of cDPP3 level to the ORBI risk score improved the discrimination and reclassification value for developing in‐hospital CS (net reclassification improvement [NRI] 0.255, *p* = 0.01).^87^ Furthermore, the addition of cDPP3 to the GRACE 2.0 score improved discrimination and reclassification of 30‐day and 1‐year mortality with NRI 0.125 and 0.347, respectively.^87^

In a substudy comparing noradrenaline and adrenaline support in 57 patients with CS, cDPP3 correlated with reduced cardiac index, lower estimated glomerular filtration rate (eGFR) and a higher Simplified Acute Physiology Score II.^151^ Similarly, in a small prospective study of 15 patients, high cDPP3 levels correlated with pulmonary hypertension, reduced stroke volume and the need for mechanical ventilation.^152^

In a large, prospective, multicentre cohort study of 4787 patients with ACS, cDPP3 on admission was predictive of the development of CS, and was superior to conventional markers such as troponin and NT‐proBNP.^87^, ^153^, ^154^ In a subgroup of 12 patients who underwent cardiac magnetic resonance imaging, cDPP3 level was associated with infarct size.^87^ Persistently elevated cDPP3 levels were associated with a roughly 10‐fold increased 30‐day mortality, and remained independently predictive after adjustment for established risk factors.^87^ Thus, cDDP3 holds promise as a marker of both CS onset and severity, although validation of these early findings is required.

## Novel biomarkers predicting mortality in cardiogenic shock

### Markers of hypoperfusion

#### Intestinal fatty acid binding protein

Found in small bowel enterocytes, intestinal fatty acid binding protein (I‐FABP) is released during intestinal hypoperfusion or congestion.^155^ In patients with CS or acute HF, elevated I‐FABP levels on admission were associated with 30‐day mortality.^101^ In acute HF, the addition of I‐FABP to a prediction model for mortality that included age and NT‐proBNP, significantly improved its accuracy.^101^, ^156^ However, I‐FABP is a non‐specific marker elevated in all forms of shock, where it is also associated with 28‐day mortality.^157^

### Markers of inflammation

#### Angiopoietin‐2

Angiopoietin‐2 (Ang‐2) is released from endothelial cells in response to cytokines, which subsequently leads to impaired endothelial integrity. It is involved in the systemic inflammatory response and regulates vascular tone, as well as capillary permeability.^158^, ^159^ In sepsis, Ang‐2 levels not only discriminate between sepsis and septic shock, but also correlate with mean arterial pressure, creatinine, PCT and the Sequential Organ Failure Assessment score.^160^ In chronic HF patients, a stepwise increase in Ang‐2 was observed with worsening New York Heart Association class, 6‐min walk test and peak oxygen consumption, but did not correlate with LV function.^161^ Moreover, elevated Ang‐2 levels are associated with increased cardiovascular mortality in the general population.^162^

In the Intra‐Aortic Balloon Pump (IABP)‐SHOCK II trial, Ang‐2 levels were predictive of 30‐day and 1‐year all‐cause mortality, independent of IABP therapy.^80^ The prognostic value of Ang‐2 levels increased as levels rose from day 1 to day 3 in patients who died within 30 days of admission. Furthermore, Ang‐2 levels correlated with cardiac power index, a strong predictor of mortality in CS.^80^, ^163^

#### Interleukin‐6

Interleukin‐6 is released during systemic inflammation and, besides its effect on vessels, has negative effects on the myocardium.^164^ In a subanalysis of 183 patients in the CardShock study, IL‐6 was found to be a reliable and early marker of 30‐day all‐cause mortality in ACS complicated by CS, with one of the highest AUCs among a range of biomarkers including NT‐proBNP and PCT. While IL‐6 is associated with clinical and biochemical signs of hypoperfusion, it lacks specificity for CS.^38^, ^39^

#### Fibroblast growth factor 23

Fibroblast growth factor 23 (FGF‐23) was first discovered in osteoblasts and osteocytes and is involved in the regulation of phosphate levels.^165^ Interestingly, FGF‐23 is predictive of MACE in both CKD and elderly patients.^166^, ^167^ *In vivo* and *in vitro* studies show adverse effects of FGF‐23 on the myocardium, with elevated FGF‐23 levels linked to adverse events in chronic HF.^168^ In CS, a rise in FGF‐23 levels is associated with increased 28‐day mortality.^114^ However, whether elevation in FGF‐23 levels reflects reduced filtration or excess myocardial production, remains unclear.^169^, ^170^ Furthermore, whether FGF‐23 plays an active role in this process or is simply a bystander marker, is also unclear.

#### Soluble suppressor of tumorigenicity 2

Suppressor of tumorigenicity 2 (ST2) is a member of the IL‐1 receptor family. In inflammation, IL‐33 signals via the ST2 pathway and provides myocardial protection.^171^ Soluble ST2 (sST2) acts as a decoy receptor blocking signalling and is associated with pro‐fibrotic cardiac remodelling and adverse outcomes in chronic HF.^172^ sST2 is elevated on day 1 after acute myocardial infarction and is inversely correlated with LV systolic function.^173^ Combining sST2 and NT‐proBNP was predictive of 30‐day all‐cause mortality and improved the prediction of outcome when added to the CardShock score.^174^ Small studies in CS found that prediction of mortality increases with time from onset of CS (*Table* *2*). Together, sST2 and bicarbonate levels predicted the requirement for renal replacement therapy after initiation of extracorporeal membrane oxygenation (ECMO).^175^ However, sST2 is not cardio‐specific, with higher levels present in sepsis, compared to those in CS or STEMI.^176^

#### Soluble urokinase‐type plasminogen activator receptor

Soluble urokinase‐type plasminogen activator receptor (suPAR) is a cleavage product of the urokinase‐type plasminogen activator receptor (uPAR) that is involved in inflammation, fibrinolysis, apoptosis, tissue remodelling, cell adhesion and migration, and may play a part in the formation of metastases.^177^, ^178^ Once cleaved into suPAR, it remains stable and is actively involved in inflammatory chemotaxis.^179^ Levels increase as part of systemic inflammation, in infection, cancer and cardiovascular disease, and are associated with increased mortality.^104^, ^180^

In a subset of 161 patients from the CardShock study, mainly with ACS, suPAR was independently predictive of a nearly six‐fold increase in 90‐day all‐cause mortality, with the addition of 12‐h suPAR levels to the CardShock risk score significantly improving mortality prediction.^103^ SuPAR levels in CS are lower than in sepsis, indicating a potential use for this marker in differentiating these two conditions. SuPAR levels correlated with markers of end‐organ dysfunction such as creatinine, eGFR, NT‐proBNP and CRP.^103^

### Predictors of acute kidney injury

As the number of organs affected by CS increases, the mortality rate also increases.^68^, ^181^ Hypoperfusion and/or congestion lead to progressive multi‐organ failure, as reflected by a rise in biomarkers of renal and liver dysfunction. Approximately a third of patients with CS develop acute kidney injury (AKI), which is associated with a poor prognosis.^68^ The most frequently used marker of AKI is creatinine, which is utilized in risk assessment systems such as the IABP‐SHOCK II score.^20^, ^182^ More sensitive markers of AKI such as Ang‐2, cystatin C, kidney injury molecule‐1 (KIM‐1), neutrophil gelatinase‐associated lipocalin (NGAL) and proenkephalin (PENK), have been evaluated in CS (see below). However, their prognostic utility has shown conflicting results across the studies.^117^, ^183^

#### Angiopoietin‐2

In the IABP‐SHOCK II substudy, Ang‐2 measured on admission was an independent predictor of AKI in patients with CS. However, Ang‐2 levels were not associated with cystatin C levels, suggesting that Ang‐2 elevation does not relate to reduced renal clearance.^80^

#### Kidney injury molecule‐1

Kidney injury molecule‐1 is a transmembrane glycoprotein expressed in kidneys and other organs. It is upregulated in the proximal renal tubules during ischaemia‐reperfusion injury.^184^, ^185^ A meta‐analysis demonstrated that urinary KIM‐1 exhibits good sensitivity and specificity for the prediction of AKI,^186^ especially following cardiopulmonary bypass.^187^ Although KIM‐1 was shown to be highly predictive of long‐term decline in renal function in healthy individuals, in the setting of CS, it does not offer additional prognostic information beyond creatinine alone.^33^, ^188^

#### Neutrophil gelatinase‐associated lipocalin

Neutrophil gelatinase‐associated lipocalin is a molecule secreted by neutrophils and renal tubular epithelial cells. It is one of the earlier markers of AKI, with plasma levels rising within 2 to 6 h after renal insult.^189^, ^190^ Collectively, the evidence regarding the predictive utility of markers such as cystatin C and NGAL in ACS and CS is conflicting.^117^, ^183^

#### Proenkephalin

Proenkephalin is a stable marker of the endogenous opioid system that has cardio‐depressive effects.^191^ High levels correlate with advanced HF, deteriorating renal function and mortality.^191^ PENK levels correlate well with eGFR and unlike NGAL, are not influenced by inflammation and predict the need for renal replacement therapy in sepsis.^192^, ^193^ In CS, both NGAL and PENK were independently associated with AKI and strongly predictive of 90‐day mortality.^117^

### Combining biomarkers in cardiogenic shock

In a study of 2002 patients with STEMI, 225 of whom developed CS, four proteins were identified as early, independent predictors of CS. These included copeptin, MR‐proADM, pro‐atrial natriuretic peptide and sST2, and as their individual levels increased, so did the risk of CS.^84^ The addition of all four markers to the ORBI score significantly improved the prediction of evolving CS, with an AUC of 0.85.

#### Cardiogenic shock 4 proteins

A recent quantitative study identified 51 proteins that are elevated in CS following STEMI, among which the four best predictors of 90‐day all‐cause mortality were liver‐type fatty acid‐binding protein (L‐FABP), beta‐2‐microglobulin (B2MG), fructose‐bisphosphate aldolase B (ALDOB), and SerpinG1 (IC1). These four proteins were thus termed ‘cardiogenic shock 4 proteins’ (CS4P).^120^ CS4P was validated in the CardShock cohort, and found to be predictive of 90‐day all‐cause mortality with an AUC of 0.83.^21^ Using the CS4P with the CardShock risk score showed a marked benefit in patient reclassification over the CardShock score alone, with a NRI of 0.49 (*p* = 0.02). Using the CS4P with the IABP‐SHOCK II risk score also showed a marked benefit in patient reclassification over the IABP‐SHOCK II risk score alone, with a NRI of 0.57 (*p* = 0.032).^120^ The success of these four proteins may lie in the combination of organ systems that they represent.^120^

Liver dysfunction is associated with increased all‐cause mortality.^181^ L‐FABP, a soluble protein found in hepatocytes and, to a lesser extent, in kidneys, intestine and lung,^194^, ^195^ plays a protective role, binding and controlling potentially cytotoxic metabolites.^196^ It is upregulated in response to tissue injury and is not CS‐specific.^120^ When measured within 24 h of CS admission, L‐FABP substantially improves the prediction of death, over clinical risk scores.^120^ ALDOB is another marker of liver damage.^120^

Beta‐2‐microglobulin, elevated in coronary artery disease, is a marker of future MACE, but has not been evaluated specifically in CS.^198^ Levels increase in response to kidney injury, which in turn is associated with increased CS mortality.^68^, ^199^

Increased levels of IC1, part of the complement pathway of inflammation,^200^ correlate with increased 30‐day mortality in patients with CS,^201^ while inhibition of IC1 in STEMI patients may provide myocardial protection from reperfusion injury.^202^

#### Cystatin C, lactate, interleukin‐6 and NT‐proBNP

In patients with CS due to STEMI who were recruited to the Culprit Lesion Only PCI Versus Multivessel PCI in Cardiogenic Shock (CULPRIT‐SHOCK) trial,^203^ integration of four out of 58 investigated proteins into a clinical risk model (CLIP score) allowed for highly accurate prediction of 30‐day all‐cause mortality with an AUC of 0.83, a finding which the authors subsequently validated in the IABP‐SHOCK II cohort.^119^ These proteins were cystatin C, lactate, IL‐6 and NT‐proBNP. The CLIP score outperformed the IABP‐SHOCK II score in predicting 30‐day mortality.

This four‐component risk score incorporates cystatin C, which appears to be superior to creatinine in predicting AKI and mortality in ACS, HF, and during critical illness.^204^, ^205^, ^206^ Given its relatively short half‐life, cystatin C levels change earlier than creatinine, providing a theoretical basis for its superiority as a marker. Furthermore, cystatin C was an independent predictor of MACE in patients with ACS, even without AKI, an effect likely mediated through its relationship with inflammation and atherosclerosis.^207^, ^208^, ^209^ However, in the IABP‐SHOCK II trial, creatinine predicted mortality more reliably than cystatin C, NGAL, KIM‐1, or eGFR.^33^ There is no consensus on which marker is best.

A recent study comparing the performance of the CLIP score in CS, septic shock, haemorrhagic shock, respiratory failure and all intensive care admissions, found that the score predicted 30‐day all‐cause mortality similarly well in all these settings.^210^

## Importance of biomarker dynamics

Temporal changes in biomarker levels can improve risk assessment. Amongst patients with elevated cDPP3, those in whom cDPP3 levels had decreased at 24 h showed a lower risk of refractory CS and death compared to those with persistently elevated cDPP3 level.^151^ Likewise, ADM and lactate normalized in surviving patients, while persistent elevations were predictive of adverse outcome. Interestingly, the prognostic usefulness of lactate to predict mortality decreased from baseline (0 h) to 5–10 days, while that of ADM increased over the same period of time.^71^

In patients with CS, IL‐6 is the predominant cytokine present in plasma. Following initiation of MCS, levels of IL‐6 declined in survivors, but continued to rise in non‐survivors.^211^ Similar trends were seen with sST2 in patients receiving LVAD support for end‐stage CS. In a small study with serial sST2 measurements, sST2 levels were elevated just before LVAD implantation and decreased substantially during MCS support, normalizing by 6 months.^212^ Another very small study showed that persistent elevation of sST2 following LVAD implantation was associated with higher 1‐year mortality.^213^

## Use of biomarkers to predict complications on mechanical circulatory support

A major limitation of MCS is the high rate of vascular and bleeding complications. In a meta‐analysis of 1866 patients on ECMO, major or significant bleeding occurred in 41%, lower limb ischaemia in 17% and stroke in 6%.^214^ While patients on microaxial LVAD support also experience high complication rates, these are lower than with ECMO.^215^

As blood flows through the tubing of MCS, the contact pathway of coagulation is activated, and coupled with inflammation, significantly increases the risk of thrombosis, necessitating anticoagulation.^216^ Additionally, MCS exposes blood to high shear stress, resulting in conformational change in von Willebrand factor. This leads to cleavage of high molecular weight multimers into smaller complexes with reduced haemostatic potential, resulting in acquired von Willebrand syndrome.^217^, ^218^ There are no formal guidelines on the optimal assessment of anticoagulation in patients on MCS, though some frameworks have been proposed.^216^

Several markers have been evaluated as predictors of bleeding in CS patients. Growth differentiation factor 15 (GDF‐15) is a transforming growth factor‐β that is upregulated in a number of conditions including cardiovascular disease. Levels are thought to rise as a consequence of tissue ischaemia and inflammation.^219^ In animals, GDF‐15 inhibits platelet integrin activation, prevents thrombosis and may serve as a marker of bleeding.^220^, ^221^ In patients with coronary artery disease, GDF‐15 predicts bleeding risk in a dose‐dependent manner, including on antiplatelet agents and anticoagulation.^131^, ^220^, ^222^ In a prospective study of patients with CS attributable to ACS who were randomized to treatment with or without IABP as a pre‐specified substudy of the IABP‐SHOCK II trial, elevated GDF‐15 levels during percutaneous coronary intervention were predictive of 30‐day all‐cause mortality, even after adjusting for other known predictors such as lactate.^93^ Adding GDF‐15 to the CardShock risk score improved the risk stratification of CS (NRI 0.18, *p* = 0.003).^223^ However, there is a lack of data supporting the use of GDF‐15 as a marker of bleeding risk during MCS.

The inflammatory cytokine Ang‐2, stored in the Weibel–Palade bodies of endothelial cells, is released in response to stimuli such as thrombin and hypoxia.^159^ In patients with CS, baseline Ang‐2 levels were not predictive of 30‐day mortality, but levels were generally observed to decline following the initiation of MCS. Furthermore, persistently elevated levels were predictive of adverse outcomes.^224^ In patients with acute myocardial infarction complicated by CS, Ang‐2 levels were associated with the development of AKI, bleeding complications, and/or the need for blood transfusion, and unaffected by IABP support.^80^ In other settings, however, Ang‐2 levels have been related to the occurrence of thrombotic events.^225^, ^226^

Galectin 3 (Gal‐3) is a galactoside‐binding protein involved in cell–cell and cell–matrix interactions, and implicated in atherosclerosis, myocardial fibrosis and HF. In a small study of patients with ACS‐related CS, low Gal‐3 levels were associated with increased 30‐day mortality.^90^ In a murine model, Gal‐3 was a marker of platelet hyperactivity and thrombus formation, and Gal‐3 inhibition achieved a potent antithrombotic effect without excessive bleeding.^227^ In patients with severe chronic HF, Gal‐3 levels fell after LVAD implantation or heart transplantation, and persistently elevated levels were associated with increased mortality.^228^

In summary, while biomarkers are predictive of adverse outcomes in patients on MCS, specific markers of excess bleeding and thrombosis risk in CS patients are lacking.

## Biomarkers as treatment targets in cardiogenic shock

Several monoclonal antibodies are being explored for immunomodulatory treatment of septic shock.^229^ As a systemic inflammatory response is involved in both septic shock and CS, there may be some treatments that benefit both conditions.

Tocilizumab is an IL‐6 targeting monoclonal antibody used in the treatment of rheumatoid arthritis and, more recently, severe acute respiratory syndrome coronavirus 2 infection.^230^ In patients presenting with out‐of‐hospital cardiac arrest (33% as a consequence of STEMI), treatment with tocilizumab resulted in a reduction of systemic inflammation and a rapid fall in myocardial injury markers in a third of patients at 6–12 h.^231^ Its efficacy in preventing CS is currently being evaluated in patients with ACS at high risk of CS, in the ongoing double‐blind, randomized controlled DOBERMANN trial.^232^

Complement activation plays an important role in the pathophysiology of shock and systemic inflammation. C1 esterase regulates the complement pathway, plasma kallikrein‐kinin and coagulation systems, and elevated levels of C1‐esterase are associated with poor prognosis in CS.^120^ In a small, randomized, double‐blind trial in which 80 patients with STEMI undergoing emergency coronary artery bypass grafting were randomized to a C1‐esterase inhibitor or placebo, complement pathway inhibition was associated with shorter intubation time, shorter hospital stay, and reduced need for inotropic support.^202^ However, another small study showed no effect of C1 esterase inhibition on mortality in patients undergoing emergency coronary artery bypass grafting for ACS.^233^ Notably however, C1 esterase inhibition was associated with reduced troponin levels.^233^

Circulating DPP3 is not only a useful biomarker in CS, but is also involved in myocardial depression. In mice, intravenous infusion of DPP3 immediately resulted in reduced myocardial contractility and impaired renal perfusion.^234^ While procizumab, a cDPP3‐antibody, almost completely normalized cardiac function and renal perfusion in a murine model of isoproterenol‐induced HF, it has not yet been tested in humans.^234^

## Limitations of current data

Research into the pathophysiology, risk stratification and management of CS faces several challenges. Firstly, heterogeneity in the definition of CS is a significant limitation. Most trials and guidelines define CS as a systolic blood pressure (SBP) ≤90 mmHg for >30 min or the need for vasopressors/inotropes to maintain SBP >90 mmHg.^235^ However, CS can also present with normotension, with a similar poor prognosis to hypotensive CS. Unfortunately, the former group is usually excluded from trials.^236^, ^237^ Recently, a consensus document from the Shock Academic Research Consortium aimed to standardize definitions and research into CS, although multiple, frequent changes in definition may lead to confusion.^2^, ^238^

Secondly, CS is a condition with diverse aetiologies, including ischaemia, primary pump failure, valvular disease, arrhythmias or following bypass surgery. While some trials focused on a single aetiology of CS (predominantly ACS), many have grouped various aetiologies together. Although similar vicious cycles of maladaptive responses lead to further deterioration in cardiac output and ischaemia, differing pathophysiological causes may likely require different treatments, with variable response rates.

A significant limitation of most biomarkers is that they are non‐specific, and seem to identify a ‘sick’ patient with high mortality, rather than a specific CS cohort, with specific risks. Accordingly, the elevation of circulating levels of most biomarkers in CS is considered to be an epiphenomenon and not pathologically causal. Some molecules (e.g. cDPP3 and IL‐6) may represent potential biotargets to modulate cardiovascular outcomes.

The effect of distinct treatment decisions based on biomarkers, such as mechanical offloading, inotropic requirements or haemofiltration, has not been well characterized thus far. Furthermore, most of the available data on biomarkers in CS stem from a number of small studies and a few larger interventional trials. The larger studies were designed to assess the effect of interventions such as IABP in CS, and not specifically to examine biomarkers. Six biomarkers (see *Table* *2*) and two scores (CS4P and CLIP score) have undergone some external validation for the prediction of mortality. These are often in heterogeneous populations using different cut‐off values compared to those in the derivation cohorts. Further, more rigorous external validation is needed before these are integrated into protocols and used in clinical practice.

Furthermore, the findings from these studies are still limited to the populations in which they were conducted. There were no differences in sST2 levels between men and women with CS receiving LVAD support, while none of the other studies reported gender differences specifically.^212^ Local demographics, ethnicity, and clinical practices may influence the usefulness of the biomarkers and require further validation. Additionally, patients from specific demographics or with certain beliefs may have been excluded from these studies, further limiting the generalizability of the biomarkers.

Most of the novel biomarkers discussed herein are not widely available, require testing in specialized laboratories, and even then, the results are not available in a timely manner. CS is a time‐critical condition, meaning that many of these markers are impractical for practical clinical use. Biomarkers that can be tested at the bedside would facilitate the rapid decision‐making required in CS patients.

Cost‐effectiveness is another crucial consideration when choosing tests for patients with CS. The usefulness of biomarkers will be determined not only by their sensitivity and specificity for predicting outcomes, but also by their added value (net reclassification) compared to current biomarkers and risk scores, taking into account the frequency of measurements and the availability of tests (including turnaround times/near‐patient options) compared to more readily available biomarkers such as creatinine and lactate. Furthermore, determining the optimal timing and frequency of measurements to best guide prognosis and cost‐effectiveness requires further study for all biomarkers.

These limitations indicate that we have not yet reached the point of establishing thresholds and timings that would allow for the incorporation of these new biomarkers into clinical use. Further research is therefore needed before any of the novel markers can be incorporated into routine use to help predict the onset of or the outcome of CS.

## Future directions

Given the high mortality and morbidity of CS and the failure of many new treatment strategies, there is a significant need to improve outcomes. Important first steps are to standardize the definition of CS used in studies, to define the underlying aetiology leading to CS, as well as the shock stage at which a potential marker is assessed. Indeed, many interventions may be effective in early (i.e. SCAI stages A‐C), but not in fully developed CS (i.e. SCAI stages D‐E).

Some novel biomarkers may have the potential to predict the onset of CS before it progresses to more severe stages. Identifying this risk early can facilitate prompt intervention, such as more invasive haemodynamic monitoring, and enable the use of early, targeted treatments. It also allows for thorough assessment and planning of escalation strategies.

New biomarkers that detect early end‐organ hypoperfusion, before routinely available blood markers indicate organ damage, could help guide interventions to optimize haemodynamic support or escalate to MCS early. Bleeding and thrombotic complications are common in CS, especially in patients on MCS. Biomarkers could also help guide anticoagulation levels and predict thrombotic or bleeding events. Minimizing these complications may improve survival, particularly in patients on MCS.

However, to use the biomarkers for decision‐making, we first need large prospective studies to evaluate biomarker trends in CS. Furthermore, research will be needed to transition the biomarker use from bench to bedside, to guide interventions and to determine whether these changes have meaningful effects on clinical outcomes.

Most studies have focused on relatively short‐term outcomes (see *Table* *2*). Patients discharged from the hospital often suffer from significant morbidity and remain at increased mortality risk, which can lead to rehospitalization.^6^ Biomarkers that predict morbidity and readmission could be useful for enabling closer follow‐up of patients post‐discharge, to achieve the best long‐term outcomes, whether that is active treatment or discussions around best supportive management.

Several studies, both observational and interventional, are in progress or planned, to evaluate existing and identify new biomarkers (*Table* *3*). The ideal biomarker should be readily available for widespread use, ideally at the point‐of‐care, and give results in a suitably rapid manner to influence care. New combinations of biomarkers need to be examined to determine whether they perform better than individual markers alone.

**Table 3:** Ongoing observational and interventional trials investigating the levels of biomarkers in patients with cardiogenic shock

Name of trial	NCT	Patient population	Country	Start‐end date
Cardiogenic Shock Integrated PHenotyping for Event Reduction (CIPHER)	NCT04323371	Patients with acute decompensated heart failure, complicated by CS	Italy	2020–2025
ECMOsorb Trial ‐ Impact of a VA‐ECMO in Combination With CytoSorb in Critically Ill Patients With Cardiogenic Shock (ECMOsorb)	NCT05027529	CS of any cause and indication for VA‐ECMO	Germany	2021–2024
Normoxemic Versus Hyperoxemic Extracorporeal Oxygenation in Patients Supported by Veino‐arterial ECMO for Cardiogenic Shock (ECMOxy)	NCT04990349	Patient supported by VA‐ECMO for CS	France	2022–2024
Genomic Determinants of Outcome in Cardiogenic Shock (Goldilocs)	NCT05728359	CS secondary to AMI or myocarditis	UK	2022–2025
Role of Candidate Proteins in Capillary Leakage During Acute Circulatory Failure	NCT05586282	Circulatory shocks (including septic shock and CS)	France	2022–2026
Influence of Enteral Microbiome on Mortality of Patients With Cardiogenic Shock	NCT06006754	CS of all aetiology	Germany	2023–2024
PRecision Ecmo in CardIogenic Shock Evaluation (PRECISE)	NCT05748860	Patients who will be commenced on VA‐ECMO (for CS or ECPR)	Australia and New Zealand	2023–2026

AMI, acute myocardial infarction; CS, cardiogenic shock; ECMO, extracorporeal membrane oxygenation; ECPR, extracorporeal cardiopulmonary resuscitation; VA‐ECMO, veno‐arterial extracorporeal membrane oxygenation.

Some biomarkers represent promising therapeutic targets. Many trials are currently searching for new targets in CS (*Table* *3*). Multiple cytokines are generated during CS, and one strategy being explored involves removing these from the circulation using filtration with ECMO, although some cytokines may have protective functions in CS (*Figure* *2*).^239^ Treatments targeting biomarkers at earlier stages of CS or pre‐shock, are needed to define the ‘sweet spot’ for personalized therapeutic interventions. Future clinical trials should also explore a biomarker‐tailored treatment approach. For example, patients with high levels of IL‐6 may be prone to a mixed distributive‐inflammatory phenotype of CS, which could respond to an anti‐inflammatory treatment. In this regard, treatment of CS with the anti‐IL‐6 monoclonal antibody tocilizumab or the cDPP3 inhibitor procizumab, appear particularly promising.

### Beyond proteomics

Working out the molecular signature of CS is not limited to focusing on proteomics.^79^, ^240^ Since activation of transcription precedes protein formation, genomics and transcriptomics may also play an important role. This approach could allow the early detection of pathological processes, such as signatures of inflammation or AKI, enabling early implementation of treatment.^241^ Upregulation of certain RNAs is associated with renal and brain protection from hypoxia which may be another potential therapeutic avenue to explore.^242^, ^243^ Additionally, it could provide insights into genetic susceptibility, including to developing complications, with implications for management.^244^

Furthermore, with the rise of ultra‐rapid genome sequencing, genomics may play a role in facilitating targeted treatments.^245^ In the future, artificial intelligence may be able to integrate multiple variables and assist with the personalization of treatment in CS.^246^

## Conclusion

A number of novel biomarkers enhance mortality prediction in CS compared to conventional markers such as lactate, with some, such as ADM and cDPP3 able even to predict the development of CS. Temporal changes in biomarkers may guide prognostication particularly well. Many biomarkers reflect end‐organ hypoperfusion or systemic inflammation, and are therefore not specific to CS. While some constitute potential therapeutic targets, the limitations of current biomarkers include lack of widespread availability, slow turnaround times, lack of specificity and limited prospective validation. This highlights the significant unmet need in CS and provides clinicians with an overview of current and novel biomarkers, thereby stimulating future research into and validation of these biomarkers. Future studies assessing novel biomarkers to predict the onset and prognosis in CS, assess the impact of interventions and act as therapeutic targets could enhance the outcome in this very high‐risk cohort.

**Conflict of interest**: F.A.W. has received support from the Foundation for Cardiovascular Research – Zurich Heart House, the Theodor und Ida Herzog‐Egli Foundation, the Lindenhof Foundation, the European Society of Cardiology, the Swiss Heart Foundation, the Fonds zur Förderung des akademischen Nachwuchses of the University of Zurich, the Medical University of Graz, the Sphingotec GmbH, the 4TEEN4 Pharmaceuticals GmbH, and the PAM Theragnostics GmbH outside this work. V.P. has received consulting fees from Johnson & Johnson and Abiomed. D.A.G. has received institutional research grants from Bayer, AstraZeneca, Werfen and Medtronic, and advisory board/steering committee roles with BMS, Janssen and Chiesi. All other authors have nothing to disclose.
